# Neuroimaging-Based Brain Age Estimation: A Promising Personalized Biomarker in Neuropsychiatry

**DOI:** 10.3390/jpm12111850

**Published:** 2022-11-05

**Authors:** Daichi Sone, Iman Beheshti

**Affiliations:** 1 Department of Psychiatry, Jikei University School of Medicine, Tokyo 105-8461, Japan; 2 Department of Human Anatomy and Cell Science, University of Manitoba, Winnipeg, MB R3E 3P5, Canada

**Keywords:** brain age, neuropsychiatric disorder, neuroimaging, machine learning

## Abstract

It is now possible to estimate an individual’s brain age via brain scans and machine-learning models. This validated technique has opened up new avenues for addressing clinical questions in neurology, and, in this review, we summarize the many clinical applications of brain-age estimation in neuropsychiatry and general populations. We first provide an introduction to typical neuroimaging modalities, feature extraction methods, and machine-learning models that have been used to develop a brain-age estimation framework. We then focus on the significant findings of the brain-age estimation technique in the field of neuropsychiatry as well as the usefulness of the technique for addressing clinical questions in neuropsychiatry. These applications may contribute to more timely and targeted neuropsychiatric therapies. Last, we discuss the practical problems and challenges described in the literature and suggest some future research directions.

## 1. Aging, Disease, and the Brain

The aging process in humans is associated with the progressive decline of various physiological and organ functions [1], and many diseases including cancer, cardiovascular disease, diabetes, and dementia are associated with aging [2]. It is not uncommon for elderly people to suffer from multiple diseases simultaneously. Since humans’ bodies change with age and as humans are living longer in several regions of the world, the aging process has become a key issue in public health, disease prevention, and treatment. Many discussions concerning the pathological meaning of aging in the context of epigenetic change, proteotoxic or oxidative stress, and telomere damage have thus been conducted [3].

The brain is also affected by aging [4]. In the early stage of life, the aging process is regarded as brain development in which the brain matures, and children usually experience an increase in their cognitive ability along with their physical growth. During late adulthood, the brain aging process has different effects, e.g., a decline of cognitive function, and advancing age is associated with neurodegeneration, particularly Alzheimer’s disease and other forms of dementia [5,6]. If the aging process of the brain could be measured precisely and accurately, the findings may have potential as biomarkers for neuropsychiatric disorders. In fact, frameworks to quantify the age of a human brain have been attempted for several decades [7]. Today, advances in medical imaging and analytical methods (especially machine learning) have allowed the calculation of an individual’s biological age from the extracted biological features [8]. The frameworks that are now used to estimate the age of an individual’s brain have the potential to provide useful, objective, and personalized biomarkers for neurological and psychiatric disorders.

## 2. Neuroimaging-Based Brain-Age Estimation

Telomere-related and epigenetics-related biomarkers have not shown sufficient predictive and deterministic value for estimating brain ages, and it has been suggested that phenotype-based estimation can generate a much closer indicator of brain age [4]. Neuroimaging is a widely available, less-invasive method to investigate the whole brain of humans, and with neuroimaging, the brain’s morphological and microstructural features can be obtained; these features are speculated to be suitable material for the estimation of the age of an individual brain. In fact, neuroimaging-based brain-age estimation has been increasingly applied to individuals with various neuropsychiatric disorders and general populations [8]. In this narrative review, we examined over 100 studies and introduce the recent findings and methodologies of this emerging technique. We conducted a search of the PubMed database in May 2022 using “brain-age estimation” and/or “brain-age prediction” as keywords, although we did not adopt rigorous systematic selection criteria of studies for this narrative review.

## 3. Theory and Methodology

### 3.1. Theory of Neuroimaging-Based Brain-Age Estimation

In 2010, Katja Franke and her peers developed a prediction model that was able to estimate a subject’s age based on brain imaging data and the use of a regression machine-learning model [9]. The output of a brain-age estimation framework has been called the “brain age-delta,” “brain predicted age difference (Brain-PAD),” “brain age gap estimation (BrainAGE),” and “brain age gap (BAG),” each of which is computed by deducting the estimated brain age from the subject’s chronological age. In this review, we refer to “brain age-delta” as the output of a brain-age estimation framework. The brain age-delta is known as a heritable biomarker for both monitoring cognitively healthy aging and identifying age-associated disorders [8]. There are three possibilities for a brain age-delta value: (i) a brain age-delta close to zero, representing normal brain aging, (ii) a positive brain age-delta (i.e., estimated brain age > chronological age), representing an older-appearing brain, and (iii) a negative brain age-delta (i.e., estimated brain age < chronological age), representing a younger-appearing brain.

**Figure 1:**
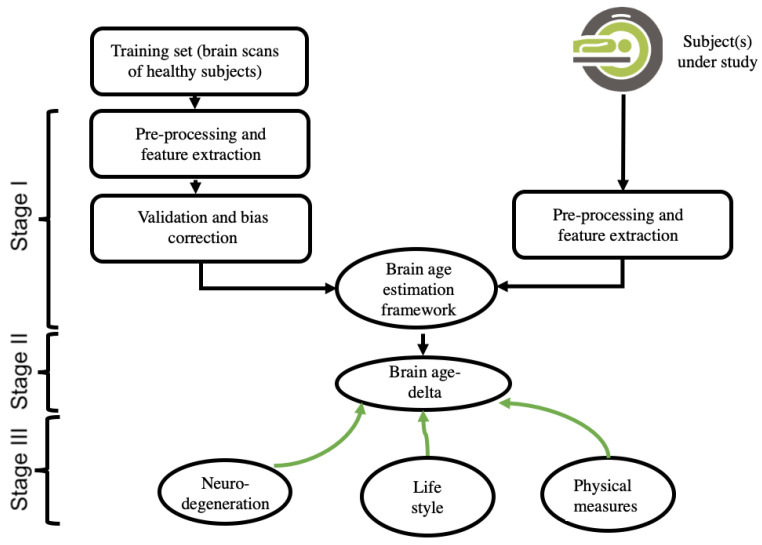
This is a figure. Schemes follow the same formatting.

A brain-age estimation study is generally composed of three main stages: (i) creating a prediction model by using extracted brain features and a regression machine-learning model, validation, and bias correction; (ii) computing the brain age and brain age-delta for the subject under study; and (iii) interpreting the results, including the use of a within-group and/or a between-groups analysis. Figure 1 depicts the pipeline of a typical brain-age estimation study.

In the literature, the typical accuracy of the prediction of brain ages is from 2 years to 10 years in terms of mean absolute error (MAE) [10,11]. The prediction accuracy in a brain-age estimation framework depends on variables such as the type of input data, the feature extraction, and reduction strategies [12] and bias adjustment techniques [13], and machine-learning models [14]. In the following subsections, we provide a general overview of these variables.

### 3.2. Input Data and Feature-Extraction Methodologies of Neuroimaging

One of the key concerns among researchers attempting to develop a brain-age estimation framework is the selection of the input data. Each modality offers unique insights into the brain. For example, fluorodeoxyglucose-positron emission tomography (FDG-PET) scans provide information about the brain’s glucose metabolism, whereas magnetic resonance imaging (MRI) data provide information about the anatomy of the brain. Among the different brain, MRI modalities such as T1-weighted MRI images (T1w MRI), T2-weighted MRI images (T2w MRI), resting-state functional (f)MRI, and fluid-attenuated inversion recovery (FLAIR), the majority of brain-age estimation studies have used T1w MRI data. The main reason for using T1w MRI is because it is more readily available than other modalities [15]. Brain age frameworks generally require a large dataset for training a prediction model, and many public neuroimaging datasets such as ADNI (https://www.adni.loni.usc.edu, accessed on 31 October 2022), PPMI (https://www.ppmi-info.org, accessed on 31 October 2022), IXI (http://brain-development.org/ixi-dataset/, accessed on 31 October 2022), and OASIS (https://www.oasis-brains.org/, accessed on 31 October 2022) have provided a great number of T1W MRI scans for research studies.

Each brain imaging modality requires a specific feature extraction strategy. The feature extraction approaches for T1w MRI data can be classified into two categories: (i) voxel-wise methods (e.g., statistical parametric mapping [SPM], http://www.fil.ion.ucl.ac.uk/spm, accessed on 31 October 2022) [8,16,17], which use gray matter (GM) and/or white matter (WM) signal intensities as brain features; and (ii) region-wise methods (e.g., FreeSurfer, http://surfer.nmr.mgh.harvard.edu/, accessed on 31 October 2022) [18], which use the subcortical and cortical and measurements of volume, surface, and thickness values as brain features. Both voxel-wise and region-wise feature extraction approaches have been widely used in T1-w MRI-driven brain-age estimation studies [19,20,21].

A direct comparison of voxel-wise and region-wise metrics as well as their integration in the accuracy of brain age has been conducted [12]. For functional MRI-driven brain-age frameworks, the extracted features can be functional connectivity (FC) measures between brain regions or intrinsic connectivity networks and voxel-wise whole-brain FC measures (e.g., FSLNets, https://fsl.fmrib.ox.ac.uk/fsl/fslwiki/FSLNets, accessed on 31 October 2022) [22,23]. In terms of the PET modality, the extracted features for estimating brain ages include measurements of brain metabolism (i.e., PET regional total glucose, aerobic glycolysis, oxygen) and cerebral blood flow [22,23]. White-matter microstructure measurements such as mean diffusivity, fractional anisotropy, axial diffusivity, and radial diffusivity have been employed as brain features for a diffusion tensor imaging (DTI)-based brain age framework [24].

### 3.3. Data Reduction, Validation, and Bias Adjustment Neuroimaging Methodologies

The ‘curse of dimensionality’ is one of the major concerns in developing a brain-age estimation framework, particularly when the number of brain features is far higher than the number of samples (e.g., in voxel-based feature extraction strategies). High-dimensional data can give rise to some substantial issues in a prediction model, such as overfitting and decreased computational efficiency. A data reduction technique that can decrease the high dimensionality of data and diminish redundant information is thus required. In the area of brain-age estimation, most studies have used the principal component analysis (PCA) strategy, which is an unsupervised learning technique [9,19]. The effect of the number of principal components on the accuracy of brain-age predictions has been investigated [9]. The number of principal components may influence the prediction accuracy in a brain age estimation framework. However, it can be adjusted to achieve maximum accuracy in the training set [9].

After a prediction model is developed, it is critical to validate the model’s prediction accuracy. Most studies in the field of brain-age estimation have used a K-fold cross-validation strategy (e.g., K = 5 or 10) to assess the prediction performance on a training set [12,14,16,21]. In the K-fold cross-validation technique, the data are randomly divided into K folds, and the learning process is repeated K times so that K-1 folds are used for training a prediction model, and the remaining fold is used as a test for each iteration. To assess the prediction accuracy, researchers generally use the coefficient of determination (R2) between the subjects’ chronological age and estimated age, the MAE, and root mean square error (RMSE) metrics.

Many brain-age estimation studies have reported age dependency on the prediction outputs, and this is considered a substantial issue in brain-age frameworks [13,21]. This bias, which could be a result of regression dilution bias, may adversely affect the predicted values and alter the interpretation of results. Several techniques have been proposed to diminish this bias (i.e., age dependency) [13,21,25]. For instance, Le and colleagues proposed using chronological age as a covariate in the statistical analyses and interpreting the results [26]. However, it should be highlighted that Le’s method is appropriate for group comparison only and not able to deliver bias-free brain age values at the individual level. A bias adjustment strategy is proposed in [21] (i.e., Cole’s method) that uses the intercept and slope of a linear regression model of estimated brain age against chronological derived from the training set. The bias-free Brain-age values in the test sets are then calculated by subtracting the intercept from the predicted brain age and dividing by the slope [21]. The most recent bias adjustment technique is suggested in [13] (i.e., Beheshti’s method) which computes offset values for test subjects on the basis of the intercept and slope of a linear regression model of brain age-delta against chronological age achieved from the training set. Then, the bias-free Brain-age values are computed by subtracting the offset values from the estimated brain-age values [13]. A direct comparison of these bias adjustment techniques has shown that Beheshti’s method greatly reduces the variance of the predicted ages, whereas Cole’s method increases it [13].

### 3.4. Machine-Learning Methodologies

One of the important steps in developing a brain-age estimation framework is choosing a regression machine-learning model. A regression model establishes a pattern between independent variables (here, brain features) and the corresponding dependent variable (a subject’s chronological age) based on the training dataset, and the model uses this pattern to predict the brain age based on unseen data (i.e., independent test datasets). The most widely used traditional regression algorithms include support vector regression (SVR) [19,23], relevance vector regression (RVR) [9], Gaussian process regression [21], an ensemble of gradient-boosted regression trees [25], and XGBoost [25]. It has been demonstrated that the type of regression algorithm used influences the prediction accuracy and the interpretation of outcomes in brain-age frameworks [14].

In addition to the traditional regression algorithms, deep learning models have become a prominent methodology in the area of brain-age estimation [11], as they can be used to develop more accurate prediction models. A major advantage of deep learning models is that they can be applied directly with 3D brain image data and incorporate feature extraction, data reduction, and prediction stages into a unified system. The main challenge of deep learning-based brain-age estimation frameworks is that this methodology requires a large dataset to train a model. In 2017, James Cole and his peers developed the first deep learning-based brain-age estimation framework, with a 3D convolutional neural network (CNN) that uses 3D gray matter and 3D white matter intensity maps as the input data [11]. Other deep learning architectures used in brain-age estimation frameworks include feed-forward neural networks, VGGNet [27], ResNet [28], U-Net [29], and an ensemble of CNN architectures [30].

## 4. Applications in Neuropsychiatry

### 4.1. Alzheimer’s Disease, Dementia, and Memory Impairment

One of the most active areas of brain-age research concerns Alzheimer’s disease (AD) and mild cognitive impairment (MCI) (Table 1) [17,23,31,32,33,34,35,36,37], because of their strong association with aging. Alzheimer’s disease is the most common cause of dementia, which is also a relevant issue in aging societies in many developed countries. The early detection of AD is important in terms of early cognitive intervention [38,39,40] as well as the future development of disease-modifying therapy [41], and neuroimaging plays key roles in ensuring accurate and early diagnoses, revealing the underlying pathophysiology, and monitoring the disease. An increased BAG in individuals with AD has been consistently reported, ranging from +2.88 to +9.29 years [17,20,37], and correlations between an increased BAG and cognitive dysfunction or white matter hyperintensity were also found [17,36]. Importantly, the predictability of progression from MCI to AD and the detectability of preclinical AD based on brain-age measures are also confirmed and would be clinically significant [31,34,37].

### 4.2. Other Neurological Diseases

Parkinson’s disease (PD) is a common neurodegenerative movement disorder characterized by the degeneration of dopaminergic neurons in the substantia nigra [55]. Overall, it seems that an increase in brain age by 2–3 years occurs in PD [20,42,43], and such an increase is associated with cognitive or motor impairment (Table 1). A comparison study between PD and AD revealed a significant increase in the BAG in AD compared to PD.

Epilepsy is also a common neurological disorder, characterized by recurrent seizures associated with abnormal electrical activity in the brain. A brain with chronic epilepsy tends to present a BAG of +4 to +8 years (Table 1) [19,44,45,46], and comorbid psychosis may further increase the BAG by several additional years [19]. Interestingly, epilepsy surgery may reduce the abnormal BAG increase, regardless of postsurgical seizure freedom [46].

Multiple sclerosis (MS) is an autoimmune disease involving damage to the myelin sheaths of the brain and spinal cord [56]. The BAG in MS is relatively high at +6.5 to 10.3 years on average (Table 1), and it is particularly higher in secondary progressive MS (+13.3 years) [47,48]. An increased BAG is also suggested to predict MS progression.

A brain-age framework has also been applied to neurological and related disorders including traumatic brain injury [49,50], pain [51,52], Prader-Willi syndrome [53], and HIV infection [54] (Table 1).

### 4.3. Schizophrenia and Psychotic Disorders

Schizophrenia is a serious psychiatric disorder presenting symptoms that include psychosis, cognitive dysfunction, and negative symptoms. Increased brain age in schizophrenia and first-episode psychosis (FEP) has been reported (Table 2) [57,58,59,60,61,62,63,64,65,66,67,68,69,70,71,72], and consistent findings of a BAG increased by approx. 3–6 years in schizophrenia have been confirmed, with possible associations with cognitive dysfunction or polygenic risk. The BAG in individuals with FEP may be lower than that in schizophrenia, and, according to longitudinal studies, an acceleration of brain aging over time is suggested in this population. The BAG is also associated with schizotypal symptoms in relatives of patients with psychosis [72]. Early medication may reduce the BAG in psychosis [71].

### 4.4. Mood Disorders

There have been several studies of brain age in mood disorders (Table 2), including bipolar affective disorder (BPAD) and major depressive disorder (MDD) [73,74,75,76,77,78,79,80,81]. Unlike schizophrenia, some studies reported no significant difference in the BAG in mood disorders [59,62,73], while others found an increase of approx. +2 to +4 years [76,77,79,80,81]. Overall, the aging abnormality in mood disorders would be mild to moderate. Two studies that focused on MDD in late life reported significantly increased brain age [76,77]. Interestingly, the BAG may be reduced by medications, such as lithium for BPAD or antidepressants for MDD [74,80].

### 4.5. Other Psychiatric Disorders

The brain age in other psychiatric disorders such as obsessive-compulsive disorder (OCD) and specific phobias has been investigated [82,83] (Table 2), and a relatively large study reported contributions of both severe mental illness and cardiometabolic disorders to an increased BAG [84].

### 4.6. Comprehensive Studies

Brain-age findings across various neuropsychiatric disorders have been obtained in comprehensive studies [85,86,87,88] (Table 2). Overall, these studies successfully identified neuropsychiatric disorders and risk factors by using brain age, and it was indicated that a multimodal imaging model may have high accuracy [88]. In particular, an investigation of a large sample (>10,000 patients and 35,000 healthy controls) revealed the effect sizes of a BAG in various conditions, which should be regarded as a reliable standard of BAG scores so far [85]. According to this study, the strongest aging of the brain is seen in dementia, followed by MS, schizophrenia, and MCI.

## 5. Applications to General Populations

Targeting a general population or individuals without neuropsychiatric diagnoses is another important topic in neuroimaging-based brain-age framework research, as it may clarify how to keep our brains healthy and avoid the risks of accelerated aging [15,16,21,89,90,91,92,93,94,95,96,97,98,99,100,101,102,103,104,105,106,107,108,109,110,111,112,113,114,115,116,117,118,119,120,121,122,123,124,125,126,127,128,129]. The most consistent significant risk factor could be diabetes (Table 3). In fact, diabetes has been consistently reported to adversely affect the aging of the brain. Alcohol consumption and smoking were also associated with an increased BAG in some studies [15,16,121,122,123,128]. Other factors that were suggested to be associated with an increased BAG include mortality, allostatic load, lung function, exposure to famine in early gestation, recidivism, chronic pain, cardiovascular risk, chemotherapy for cancer, lead exposure in childhood, hypertension, premature birth, male sex, worry and rumination, neighborhood disadvantage, sleep apnea, obesity, and physical strength (Table 3).

In addition, several studies reported potentially protective factors associated with a reduced BAG: long-term meditation (−7.5 years), music composition (approx. −4 years), physical activity, taking ibuprofen, and life satisfaction (Table 3). Interestingly, it has been observed in more than one study that childbirth decreases the BAG in women, not only during the postpartum period but also in later life [100].

Thus, although the studies are diverse in terms of the methodologies used and the targeted factors, cumulative evidence will further expand our knowledge of how to improve the aging process of human brains. The strong and consistent risk for brain aging appears to be diabetes, followed by alcohol consumption. Other lifestyle-related risk factors, e.g., smoking or hypertension, may also be harmful but less consistent. Regarding beneficial effects, though the research focuses were diverse across studies, physical, mental, or creative activities may likely improve our brain age. It is unclear whether neuropsychiatric disorders, particularly dementia, could be prevented by improving brain aging. Further research is necessary to obtain real-world evidence regarding the utility of brain-age studies for this question.

## 6. Strengths, Controversies, and Future Direction

As described above, a neuroimaging-based brain-age estimation can provide a reliable neuropsychiatric biomarker at the single-subject level. In addition, brain MRI—particularly T1-weighted structural MRI—is a widely available examination in most countries, which may support easier and wider clinical applications of brain-age analyses. Given that many studies have successfully used public databases to build a brain-age prediction model, the reproducibility and external validity of a brain-age model should be at an acceptable level. Thus, the strengths of brain age as a biomarker would be its use as a single-subject-level marker, widely available examination, and acceptable reproducibility. The processing of MRI scans, including normalization and machine-learning analysis, may require advanced techniques and could be a possible barrier for most facilities, but currently, there are several public tools, e.g., BARACUS (https://github.com/BIDS-Apps/baracus, accessed on 31 October 2022) and brainageR (https://github.com/james-cole/brainageR, accessed on 31 October 2022) which would help us apply a brain age model to the patients.

Controversies and limitations of the use of brain ages have also been suggested, particularly for the interpretation of study results. As reviewed herein, there is a level of overlapping of BAG scores across various disorders, which might limit the usefulness of the brain age for differential diagnoses in clinical analyses. It was also reported that individual variations in brain age were associated with early-life factors rather than longitudinal changes [119]. Moreover, the methodology is quite diverse in terms of imaging modalities, processing, and choice of machine-learning algorithm, and there is no established consensus about the optimal protocol for determining brain ages. Future studies should address these controversies and limitations.

In conclusion, neuroimaging-based brain-age estimation has been widely and increasingly researched for over 10 years, and many studies revealed its usefulness for neuropsychiatry. Considering the utility, availability, and reproducibility of neuroimaging-based brain-age estimations for single patients, brain age can be expected to become a useful personalized biomarker in neuropsychiatry.

## Figures and Tables

**Table 1:** Neuroimaging-based brain age studies for dementia, cognitive impairment, and other neurological disorders.

First Author [ref.]	Year	Cohort	Imaging Modality	ML Algorithm	Main Findings
**Alzheimer’s Disease and Cognitive Impairment**					
Gaser [31]	2013	133 pMCI, 62 sMCI	T1WI	RVR	BAG predicts conversion to AD, 10% greater risk of developing AD by each 1 additional yr of BAG
Lowe [32]	2016	150 AD, 112 pMCI, 36 sMCI, 107HC	T1WI	RVR	Effect of APOEe4 on BrainAGE changing rates over time
Beheshti [17]	2018	147 AD, 112 pMCI, 102 sMCI, 146 HCs	T1WI	SVR	BAG: +5.36 yr in AD, +3.15 yr in pMCI, +2.38 yr in sMCI. Correlation with cognitive function
Wang [33]	2019	3688 people (middle age to elderly)	T1WI	CNN	BAG: related to incident dementia
Mohajer [35]	2020	48 AD, 222 MCI, 60 HCs	T1WI	SVR	BAG was elevated in MCI and AD but was not associated with sleep-disordered breathing.
Ly [34]	2020	74 AD, 283 MCI, 51 preclinical AD, 83 HCs	T1WI	GPR	BAG differentiated cognitively unimpaired Amyloid (+) from Amyloid (−).
Beheshti [23]	2021	292 AD, 440 MCI, 548 HCs	FDG-PET	SVR	Younger BAG in females than in males in HCs group but not in MCI or AD groups
Habes [36]	2021	1932 MCI/AD, 8284 HCs	T1WI	RBF-kernel	BAG associated with WMH as well as cognitive function
**Parkinson’s disease**					
Beheshti [20]	2020	160 PD, 129 AD, 839 HCs	T1WI	SVR	GM-based BAG: +1.50 yr in PD, +9.29 yr in AD. WM-based BAG: +2.47 yr in PD, +8.85 yr in AD. WM-based BAG > GM-based BAG in PD
Eickhoff [42]	2021	372 PD, 172 HCs	T1WI	SVR	BAG: +2.9 yr in PD. Associated with disease duration and cognitive and motor impairment.
Charisse [43]	2022	83 PD-NC, 78 PD-MCI, 17 PD-D, 84 HCs	T1WI	SVR	RBA: +2.38 yr in PD-NC, +1.90 yr in PD-MCI, +3.52 yr in PD-D. Associated with attention deficits and working memory
Epilepsy					
Pardoe [44]	2017	42 new FE, 94 refractory FE, 74 HCs	T1WI	GPR	BAG: +4.5yr in refractory FE, no significance in new FE
Hwang [45]	2020	104 TLE, 151 HCs	T1WI, fMRI	SVR	T1-based BAG: +6.6 yr in TLE. fMRI-based BAG: +8.3 yr in TLE Association with clinical data
Sone [19]	2021	318 epilepsy, 1,196 HCs	T1WI	SVR	BAG: >+4 yr in all types of epilepsies, +10.9 yr in TLE with psychosis
de Bézenac [46]	2022	48 TLE, 37 HCs	T1WI	GPR	BAG: +7.97 yr in TLE, postsurgical reduction of BAG
**Multiple sclerosis**					
Cole [47]	2020	1204 MS/CIS, 150 HCs	T1WI	GPR	BAG: +10.3 yr in MS, +13.3 yr in SPMS, predictive value for progression
Jacobs [48]	2021	179 MS	T1WI	GPR	BAG: +6.54 yr in MS, associated with a physical disability
**Traumatic brain injury**					
Gan [49]	2021	116 mTBI, 63 HCs	DTI	RVR	BAG: +2.59 yr in mTBI, associated with post-concussion complaints
Hellstrom [50]	2021	123 mTBI	T1WI, DTI	XGBoost	No significant difference in BAG between APOEe4 carriers and non-carriers after mTBI
**Pain**					
Yu [51]	2021	31 CLBP, 32 HCs	T1WI	GPR	Discrepancy in BAG between HCs and CLBP was greater in older individuals
Hung [52]	2022	45 TN, 52 OA, 50 CLBP, 812 HCs	T1WI	GPR	BAG: +6.48 yr in TN, +9.80 yr in OA, no significance in BP. Female-driven elevation in BAG
**Others**					
Azor [53]	2019	20 PWS, 40 HCs	T1WI	GPR	BAG: +7.24 yr in PWS, Not associated with IQ, hormonal or psychotropic medications, or abnormal behaviors
Cole [54]	2017	162 HIV(+), 105 HIV(−)	T1WI	GPR	BAG: +2.15 yr in HIV(+), associated with cognitive performance

AD: Alzheimer’s disease, BAG: brain age gap, CIS: clinically isolated syndrome, CLBP: chronic lower back pain, CNN: convolutional neural network, DTI: diffusion tensor imaging, FDG-PET: 18F-fluorodeoxyglucose PET, FE: focal epilepsy, fMRI: functional MRI, GPR: Gaussian process regression, HCs: healthy controls, ML: machine learning, MS: multiple sclerosis, mTBI: mild traumatic brain injury, NC: normal cognition, OA: osteoarthritis, PD: Parkinson disease, PD-D: PD with dementia, pMCI: progressive mild cognitive impairment, PWS: Prader-Willi syndrome, RBF: radial basis function, RVR: relevance vector regression, sMCI: stable mild cognitive impairment, SPMS: secondary progressive MS, SVR: support vector regression, T1WI: T1-weighted image, TLE: temporal lobe epilepsy, TN: trigeminal neuralgia.

**Table 2:** Neuroimaging-based brain age studies for psychiatric disorders.

First Author [ref.]	Year	Cohort	Imaging Modality	ML Algorithm	Main Findings
**Schizophrenia and Psychosis**					
Koutsouleris [57]	2014	141 SZ, 104 MDD, 57B PD, 89 ARMS, 127 HCs	T1WI	SVR	BAG: +5.5 yr in SZ, +4.0 yr in MDD, +3.1 yr in BPD, +1.7 yr in ARMS.
Schnack [58]	2016	341 SZ, 386 HCs	T1WI	SVR	BAG: +3.36 yr in SZ, acceleration just after illness onset
Nenadic [59]	2017	45 SZ, 22 BPAD, 70 HCs	T1WI	RVR	BAG: +2.56 yr in SZ, no significance in BPAD
Kolenic [60]	2018	120 FEP, 114 HCs	T1WI	RVR	BAG: +2.64 yr in FES, associated with obesity
Hajek [62]	2019	43 FES, 43 HCs, 96 offspring of BPAD (48 affected, 48 unaffected), 60 HCs	T1WI	RVR	BAG: +2.64 yr in FES, no significance in early BPAD
Chung [61]	2019	476 CHR	N/A	N/A	BAG predicts conversion to psychosis in a univariate analysis but not in a multivariate analysis
Shahab [63]	2019	81 SZ, 53 BPAD, 91 HCs	T1WI, DTI	RF	BAG: +7.8–8.2 yr in SZ, no significance in BPAD
Kuo [64]	2020	26 SZ, 30 MDD, 19AD, 109 HCs	T1WI	LASSO, ICA	BAG: +5.69 yr in SCZ, +3.25 yr in AD, no significance in MDD. Association with large-scale structural covariance network
Tønnesen [65]	2020	668 SZ, 185 BPAD, 990 HCs	DTI	XGBoost	Increased BAG in SZ (Cohen’s d = −0.29) and BPAD (Cohen’s d = 0.18)
Lee [66]	2021	90 SZ, 200 HCs, 76 SZ, 87 HCs	T1WI	OLS, Ridge, LASSO, Elastic-Net, SVR, RVR	BAG: +3.8–5.2yr in SZ cohort 1, +4.5–11.7 yr in SZ cohort 2. Algorithm choice can be a cause of inter-study variability.
Lieslehto [67]	2021	29 SZ, 61 HCs	T1WI	SVR	BAG: +1.3 yr at baseline, +7.7 yr at follow-up in SZ. It was suggested that BA captured treatment-related and global brain alterations.
McWhinney [68]	2021	183FEP, 155 HCs	T1WI	RVR	BAG: +3.39 yr in FEP at baseline, longitudinal worsening was associated with clinical outcomes or higher baseline BMI
Teeuw [69]	2021	193 SZ, 218 HCs	T1WI	SVR	BAG: correlation with polygenic risk, no correlation with epigenetic aging
Wang [70]	2021	166 SZ, 107 HCs	DTI	RF	BAG: +5.903 in SZ >30 yrs old. Association with working memory and processing speed
Xi [71]	2021	60 FES, 60 HCs	DTI	RVR	BAG: +4.932 yr in FES, +2.718. Decreased BAG after early medication
Demro [72]	2022	163 psychosis, 103 relatives, 66 HCs	T1WI	SVR/RF	BAG increase in psychosis more than HCs or relatives. Associated with cognition or schizotypal symptoms in relatives
**Mood disorders**					
Bestteher [73]	2019	38 MDD, 40 HCs	T1WI	RVR	BAG: no significant change in MDD
Van Gestel [74]	2019	84 BPAD, 45 HCs	T1WI	RVR	BAG: +4.28 yr in BPAD without Li treatment, no significance in BPAD with Li treatment or HCs
de Nooij [75]	2019	283AYA	T1WI	RVR	Reduction of BAG in young high-risk individuals who developed a mood disorder over 2-yr follow-up
Christman [76]	2020	76 MDD (middle-age), 118 MDD (elderly), 130 HCs	T1WI	CNN	BAG: +3.69 yrs in geriatric MDD, no increase in mid-life MDD. Associated with cognitive and functional deficits in elderly
Ahmed [77]	2021	95 late-life depression	T1WI	CNN	BAG: +4.36 yrs in late-life depression. Not associated with treatment response.
Ballester [78]	2021	160 MDD, 111 HCs	T1WI	GPR	BAG: higher in older MDD than in younger MDD, associated with BMI in MDD, not associated with treatment response
Han [79]	2021	2675 MDD, 4314 HCs	T1WI	Ridge regression	BAG: +1.08 yr in MDD with no specific association with clinical characteristics
Han [80]	2021	220 MDD/Anxiety, 65 HCs	T1WI	Ridge regression	BAG: +2.78 yr in MDD, +2.91 yr in Anxiety. Association with somatic symptoms (+4.21 yr) and antidepressant use (−2.53 yr)
Dunlop [81]	2021	109 MDD, 710 HCs	fMRI	SVR	BAG: +2.11 yr in MDD, associated with impulsivity and symptom severity
**Others**					
Liu [82]	2022	90 OCD, 106 HCs	T1WI	GPR	BAP: +0.826 yr in OCD, associated with disease duration
Niu [83]	2022	70 SP, 77 SAD, 70 MDD, 44 PTSD, 48 ODD, 81 ADHD	T1WI	Ridge regression	Multidimensional brain-age index is sensitive to distinct regional change patterns
Ryan [84]	2022	1618 SMI, 11,849 HCs	DTI	RF, gradient boosting regression, LASSO	Additive effect of SMI and cardiometabolic disorders on brain aging, the greater effect of SMI than CMD
**Comprehensive**					
Kaufmann [85]	2019	10,141 patients, 35,474 HCs	T1WI	XGBoost	BAG: d = +1.03 in dementia, +0.41 in MCI, +0.10 in MDD, +0.74 in MS, +0.29 in BPAD, +0.51 in SZ, +0.06 in ADHD, +0.07 in ASD
Bashyam [86]	2020	353 AD, 833 MCI, 387 SZ, 12,689 HCs	T1WI	CNN	Successful discrimination for neuropsychiatric disorders
Kolbeinsson [87]	2020	12,196 people who had not been stratified for health	T1WI	CNN	Identified risk factors, e.g., MS, diabetes, and beneficial factors, e.g., physical strength
Rokicki [88]	2021	54 AD, 90 MCI, 56 SCI, 159 SZ, 135 BPAD, 750 HCs	T1WI, T2WI, ASL	RF	Highest accuracy by multimodal imaging model

AD: Alzheimer’s disease, ARMS: at-risk mental state, ASL: arterial spin labeling, AYA: adolescence and young adult, BAG: brain age gap, BPAD: bipolar affective disorder, BPD: borderline personality disorder, CHR: clinical high-risk state for psychosis, CMD; cardiometabolic disease, CNN: convolutional neural network, DTI: diffusion tensor imaging, FEP: first episode psychosis, FES: first-episode schizophrenia, fMRI: functional MRI, GPR: Gaussian process regression, HCs: healthy controls, ICA: independent component analysis, MDD: major depressive disorder, ML: machine learning, OCD: obsessive-compulsive disorder, ODD: oppositional defiant disorder, OLS: ordinary least squares, PTSD: posttraumatic stress disorder, RF: random forest, RVR: relevance vector regression, SAD: social anxiety disorder, SMI: severe mental illness, SP: specific phobias, SVR: support vector regression, SZ: schizophrenia, T1WI: T1-weighted image, T2WI: T2-weighted image.

**Table 3:** Neuroimaging-based brain age studies for general populations or those without neuropsychiatric diagnoses.

First Author [ref.]	Year	Cohort	Imaging Modality	ML Algorithm	Main Findings
Franke [89]	2013	185 people	T1WI	RVR	BAG: +4.6 yr in T2DM, Acceleration by +0.2 yr per year
Franke [90]	2014	228 elderly	T1WI	RVR	BAG associated with health markers with gender-specific pattern
Franke [91]	2015	8 women	T1WI	RVR	BAG changes during the course of the menstrual cycle
Luders [92]	2016	50 LTM, 50 HCs	T1WI	RVR	BAG: −7.5 yr in LTM
Cole [21]	2018	669 people	T1WI	GPR	Higher BAG was associated with weaker grip strength, poorer lung function, slower walking speed, lower fluid intelligence, higher allostatic load, and increased mortality risk.
Franke [93]	2018	118 elderly	T1WI	RVR	BAG: +4.3 yr in males whose mothers were exposed to famine in early gestation
Hatton [94]	2018	359 men	T1WI	SVR	BAG associated with negative fateful life events in midlife
Kiehl [95]	2018	1332 incarcerated males	T1WI	ICA	Brain age predicts recidivism, particularly when combined with other data.
Le [96]	2018	20 healthy people	T1WI	SVR	BAG: −1.15 or −1.18 yr by taking ibuprofen
Luders [97]	2018	14 healthy women after childbirth	T1WI	RVR	Brain age became younger in late postpartum by 5.4 yr.
Rogenmoser [98]	2018	42 pro-musician, 45 amateurs, 38HCs	T1WI	RVR	BAG: −3.70 to −4.51 yr in musicians
Scheller [99]	2018	34 elderly	T1WI	RVR	interaction of BAG and APOE variants, suggesting a compensation mechanism in the elderly
de Lange [100]	2019	12,021 women	T1WI	XGBoost	BAG decrease with the number of previous childbirths
Cruz-Almeida [101]	2019	47 elderly	T1WI	GPR	Increased BAG in elderly with chronic pain
Cole [15]	2020	14,701 people	T1WI, FLAIR, T2*, DTI, fMRI	LASSO	BAS associated with stroke history, diabetes, smoking, alcohol, and cognitive measures
de Lange [102]	2020	473 people	T1WI, DTI, fMRI	XGBoost	Associated with cardiovascular risk
de Lange [103]	2020	19,787 women	T1WI	XGBoost	BAG decrease with the number of previous childbirths. Involvement of brain subcortical regions
Henneghan [104]	2020	43 breast cancer with chemotherapy, 50 HCs	T1WI	SVR/RF	Trend-level increase on BAG after chemotherapy for breast cancer
Reuben [105]	2020	564 people at 45 yr	T1WI	SVR/RF	BAG: +0.77 yr in those who had lead exposure in childhood
Seidel [106]	2020	20 sepsis survivors with cognitive deficits, 44 HCs	T1WI	Kernel regression	BAG: +4.5 yr in sepsis survivor, associated with the severity of dyscognition
Anaturk [107]	2021	537 elderly	T1WI, DTI, FLAIR	XGBoost	Relationship with cumulative lifestyle measures independent of cognitive age
Bittner [108]	2021	622 elderly	T1WI	RVR	BAG: +5.04 months by combined lifestyle risk, +0.6 month by smoking, −0.55 month by physical activity
Cherbuin [109]	2021	335 middle age, 351 elderly	T1WI	RVR	BAG: +51.1–65.7days by every additional 10-mmHg increase in BP
Dunas [110]	2021	351 people	T1WI, DTI, fMRI	OLS, BRR, LASSO, ENET, SVR, RVR, GPR	BAG associated with current and past physical fitness and cognitive ability
Elliott [111]	2021	869 middle-age	T1WI	SVR/RF	Associated with cognitive function, impaired brain health at age 3, and other signs of aging
Hedderich [112]	2021	101 premature-born adults, 111 full-term controls	T1WI	RVR	BAG: +1.4 yr in premature-born adults, associated with low gestational age, low birth weight, and increased neonatal treatment intensity
Karim [113]	2021	78 older adults	T1WI, T2WI, FLAIR	GPR	BAG associated with male sex, worry, and rumination
Rakesh [114]	2021	166 adolescents	T1WI	SVR	increased BAG by neighborhood disadvantage, modulated by effortful control
Rosemann [115]	2021	169 elderly	T1WI	GPR	No association with age-related hearing loss
Salih [116]	2021	15,335 HCs	DTI	Bayesian ridge regression	Limbic tract-based BAG was most accurate and associated with daily life factors. Two SNPs were associated with BAG.
Sanders [117]	2021	122 elderly	T1WI	XGBoost	BAG decrease in more physically active women but not men
Subramaniapillai [118]	2021	1067 elderly	T1WI	Elastic net regression	Brain age was more associated with AD risk factors in women than in men.
Vidal-Pineiro [119]	2021	6950 people	T1WI	LASSO, XGBoost	No association between cross-sectional brain age and longitudinal change. Association with congenital factors, suggesting a lifelong influence on brain structure from early life
Weihs [120]	2021	690 people	T1WI	OLS	Brain age associated with AHI and ODI in PSG data
Angebrandt [121]	2022	240 HCs, 231 HCs (middle age)	T1WI	SVR/RF	Dose-dependent relation between 90-day alcohol consumption and BAG
Beck [122]	2022	790 healthy people	T1WI, DTI	XGBoost	T1-based BAG: associated with sBP, smoking, pulse, and CRP. DTI-based BAG: associated with phosphate, MCV
Bourassa [123]	2022	910 people (midlife)	T1WI	SVR/RF	BAG in midlife is associated with smoking, obesity, and psychological problems during adolescence.
Giannakopoulos [124]	2022	80 elderly	T1WI	RVR	BAG predicted a decrease in executive function over time.
Linli [125]	2022	33,293 people	T1WI	XGBoost	BAG: +1.19 yr in active regular smokers, associated with the amount of smoking
Sone [16]	2022	773 elderly	T1WI	SVR	BAG: associated with life satisfaction, alcohol use, and diabetes
Vaughan [126]	2022	57 elderly	T1WI	GPR	BAG: associated with leg strength, moderating the relationship between strength and mobility
Wang [127]	2022	165 elderly	T1WI	RVR	BAG: associated with female gender, higher education but not with APOE-e4 or family history of dementia
Whistel [128]	2022	712 people	T1WI	SVR	Association of BAG in mid- to late-life with heavier smoking and alcohol consumption in early mid-life
Zheng [129]	2022	1676 HCs	T1WI	RBF-kernel	BAG associated with worse cognitive outcomes over time

BAG: brain age gap, CNN: convolutional neural network, DTI: diffusion tensor imaging, ENET: efficient neural network, fMRI: functional MRI, GPR: Gaussian process regression, HCs: healthy controls, ICA: independent component analysis, LTM: long-term meditation practitioner, ML: machine learning, OLS: ordinary least squares, RBF: radial basis function, RF: random forest, RVR: relevance vector regression, SVR: support vector regression, T1WI: T1-weighted image, T2DM: type 2 diabetes mellitus, T2WI: T2-weighted image.

## Data Availability

Not applicable.
